# Effects of Hypertension and Exercise on Cardiac Proteome Remodelling

**DOI:** 10.1155/2014/634132

**Published:** 2014-04-27

**Authors:** Bernardo A. Petriz, Octavio L. Franco

**Affiliations:** Centro de Análises Proteômicas e Bioquímicas, Programa de Pós-Graduação em Ciências Genômicas e Biotecnologia, Universidade Católica de Brasília SGAN, Quadra 916, Módulo B, Avenida W5 Norte, 70.790-160 Brasília, DF, Brazil

## Abstract

Left ventricle hypertrophy is a common outcome of pressure overload stimulus closely associated with hypertension. This process is triggered by adverse molecular signalling, gene expression, and proteome alteration. Proteomic research has revealed that several molecular targets are associated with pathologic cardiac hypertrophy, including angiotensin II, endothelin-1 and isoproterenol. Several metabolic, contractile, and stress-related proteins are shown to be altered in cardiac hypertrophy derived by hypertension. On the other hand, exercise is a nonpharmacologic agent used for hypertension treatment, where cardiac hypertrophy induced by exercise training is characterized by improvement in cardiac function and resistance against ischemic insult. Despite the scarcity of proteomic research performed with exercise, healthy and pathologic heart proteomes are shown to be modulated in a completely different way. Hence, the altered proteome induced by exercise is mostly associated with cardioprotective aspects such as contractile and metabolic improvement and physiologic cardiac hypertrophy. The present review, therefore, describes relevant studies involving the molecular characteristics and alterations from hypertensive-induced and exercise-induced hypertrophy, as well as the main proteomic research performed in this field. Furthermore, proteomic research into the effect of hypertension on other target-demerged organs is examined.

## 1. Introduction

Hypertension is the main risk factor for cardiovascular diseases, which include stroke, coronary artery disease (CAD), and heart failure (HF) leading to ~1.8 million deaths worldwide every year [1]. Moreover, essential hypertension results from the interaction of pathological mechanisms, environmental factors, and a complex genome background [2]. Cardiac pathological hypertrophy is one of the main phenotype adaptations to hypertension. Complex molecular signalling marks this process, which is transcripted to an altered cardiac proteome. Pressure overload cardiac hypertrophy is thus often marked by dysfunction within cardiac function, which, over time, may turn into HF [3, 4].

The pathogenesis of hypertension and its pathophysiology have been widely investigated by several genomic approaches, which include analysis of candidate genes and high-throughput genetic mapping such as complex genome-wide scans [5, 6]. These strategies have also been integrated with functional physiological genomics to better understand the physiological responses resulting from gene expression and their biological interactions [7, 8]. To date, proteomic strategies have been used as a complementary tool into the investigation of the pathophysiological effects of hypertension rather than its pathogenesis.

Left ventricle hypertrophy is one of the main outcomes of pressure overload stimulus [9, 10]. This phenotype modification is driven by a complex modulation within the cardiac proteome that is still being widely investigated, since the molecular mechanism underlying this process is still not fully elucidated. Despite some morphological similarities, pathological and physiological cardiac hypertrophies are characterized by a distinct genome and proteome profile [11–13]. Moreover, it has been suggested that exercise stimulus may reduce the onset of pathological cardiac hypertrophy in hypertension, being also indicated to attenuate cardiac maladaptation thought the systematic reduction in blood pressure [14–18]. However, the effect of exercise on the hypertensive myocardium lacks more experimental and comparative proteomic data. This review therefore provides an overview of proteomic research into cardiac proteome remodelling in hypertension and exercise stimulus.

## 2. An Overview of Hypertension and Cardiovascular Diseases

Hypertension is a multifactor disease characterized by chronic elevation in blood pressure to levels equal to or above 140 mmHg systolic blood pressure (SBP) and above 90 mmHg of diastolic blood pressure (DBP) [1]. Considered a worldwide epidemic disease, hypertension is the main risk factor for cardiovascular disease [19], being epidemiologically closely associated with metabolic diseases such as obesity and diabetes [20]. Cardiovascular disease leads to ~17 millions of death per year, and, from this total, it is reported that high blood pressure is estimated to cause more than half of these deaths (over 9 million deaths every year), making it also the main risk factor in the global disease burden [21]. Well-known causes of the pathogenesis of hypertension account for approximately 5% of the cases; these involve alteration in renal salt-water homeostasis, hyperstimulation of the sympathetic nervous system, hormone dysfunction, and single gene mutation [2, 22]. Thus, the development of hypertension is attributed to multifactorial and unknown factors [2]. Indeed, the pathogenesis of essential hypertension is most likely to result from the association of several pathophysiological stimuli (e.g., obesity and diabetes) with environmental factors (e.g., diet, lifestyle, tobacco, and alcohol abuse) and genetic background [23], with hereditability estimated at 15–40% [22].

Hypertension and other pathologies, such as obesity and diabetes, together with environmental factors such as physical inactivity, diet (e.g., hypercaloric and alcohol abuse), and tobacco are likely to enhance cardiac insults [24]. These risk factors may lead to vascular dysfunctions (e.g., dysfunction in endothelial vasodilation and artery stiffness) which, if not treated, progress to cardiac damage [25]. Moreover, systemic high blood pressure leads to several impairments in cardiac apparatus, especially in relation to cardiac hypertrophy (described in the following topic). The chronic overload on the myocardium is associated with the development of heart dilation and contraction impairment [11]. When it results from hypertension or cardiac congenital pathology, cardiac hypertrophy may progress into heart failure and it can be an independent risk factor for other cardiac conditions such as myocardial infarction and arrhythmia [4, 26, 27].

Hence, the identification of the molecular mechanisms involved in cardiac hypertrophy in response to pressure overload is of prime importance to understand the pathophysiology of hypertension for the myocardium and for transition to heart failure. Moreover, the investigation of the distinct molecular regulation of pathological and physiological hypertrophy (e.g., in response to exercise stimuli) may also contribute to identifying new therapeutic targets and to a better understanding of how exercise may prevent and attenuate pathological stimuli such as hypertension.

## 3. A Brief View of Cardiac Remodelling: Pathological versus Physiological Stimuli 

Cardiac enlargement occurs mainly due to an increase in myocyte size, which is triggered by several events, including increased functional load on myocyte, activation of signalling pathways and gene expression, upregulation of protein synthesis, and formation of novel sarcomeric units [11]. Moreover, this process seems to be triggered by a mechanosensing mechanism in cardiac myocytes through stretch-sensitive ion channels, growth factor receptors, and *G*-protein-coupled receptors, linking stress and pressure overload stimulus to gene regulation and protein synthesis [3, 11].

**Figure 1:**
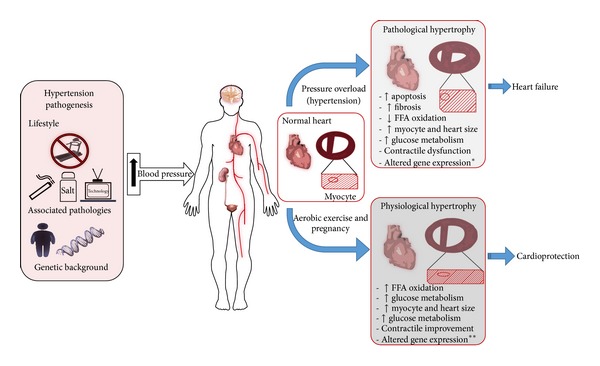
Pathologic and physiologic cardiac hypertrophy.  Figure 1 sums the factors associated with hypertension pathogenesis and its effect on some target organs (e.g., brain, kidney, and arterioles: highlighted) and cardiovascular system. Moreover, differences in cardiac hypertrophy, heart transversal session, and cardiomyocyte are presented between pathologic and physiologic hypertrophy, followed by distinct physiologic and molecular regulations. Distinct molecular regulation between pathologic and physiologic cardiac hypertrophy is associated with the development of cardiac dysfunction* or cardiac improvement**.

These molecular mechanisms are responsible for cardiac growth, a natural physiologic process, seen in the postnatal period until the heart reaches its natural size in adulthood [3]. Cardiac remodelling may also occur in response to external stimulus, which promotes heart hypertrophy such as pregnancy [28, 29] and exercise [30, 31] or as an outcome of pressure overload (e.g., aortic stenosis and systemic blood pressure) and cardiomyopathies (e.g., mutations in sarcomeric genes and associated diseases) leading to pathological cardiac hypertrophy [11]. Moreover, physiologic and pathologic cardiac hypertrophy display a distinct molecular signature resulting in a distinct cardiac phenotype [11, 32]. While physiologic hypertrophy is associated with improved cardiac function, pathologic hypertrophy is often associated with myocyte loss, fibrosis, alteration in myocyte metabolism (shift from fatty acid oxidation to glucose metabolism), and cardiac dysfunction [3, 11]. Moreover, in contrast to physiologic hypertrophy, pathological cardiac remodelling is characterized by an irreversible phenotype status [11, 33]. For a detailed view of the molecular mechanisms underlying cardiac hypertrophy see [3, 11, 34, 35].  Figure 1 presents the main alterations in heart and myocyte morphology due to pathological and physiological stimuli.

## 4. Hypertension and Pressure Overload Factors for the Cardiac Proteome

Rapid advances in the genomic field have led to a large amount of data in hypertension research, ranging from the analysis of several candidate genes to high-throughput genetic mapping (e.g., complex genome-wide scans) [5, 6]. It has been stated that the genomic approach is likely to investigate the pathogenesis of hypertension rather than its pathophysiology [36]. Moreover, functional genomic analysis and, more recently, proteomics, have been widely used to better understand the pathophysiology of hypertension. In this regard, the great advance of proteomics, as a postgenomic tool, is its ability to identify gene products, including posttranslational modifications (PTM), and further investigate the expression of these protein species for phenotype and physiological responses [37].

Pressure and stress overload lead to transcriptome regulation and triggering changes in the cardiac proteome [38]. Differences in the cardiac 2-DE proteome pattern between nonhypertensive (Wistar-Kyoto rats) and spontaneously hypertensive rats (SHR) [39] support this, while several other studies show modulations in the heart proteome followed by pressure overload hypertrophy [40]. Although heart adaptation to pressure overload is widely adverse, this molecular signalling dictates asymptomatic phenotype modulations, which over time affect cardiac structure (e.g., LV hypertrophy) and function (e.g., contractile impairment) and often evolve into heart failure [4] Figure 1.

**Figure 2:**
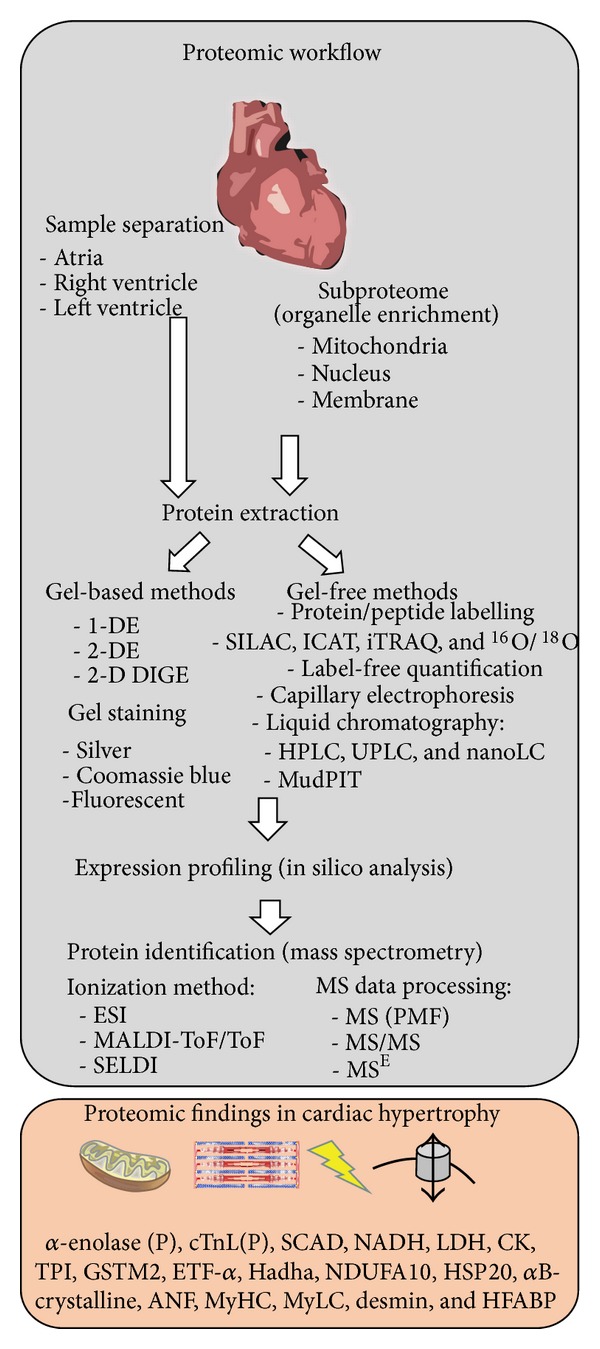
Workflow in cardiac proteome research. Figure 2 presents an overview of proteomic tools that may be used in cardiac proteome research. Starting by samples separation where heart tissue may be separated according to the research interest, followed by total protein extraction or subproteome profiling (e.g., organelle enrichment). Moreover, after protein extraction, several proteomic tools (e.g., gel-based and gel-free) may be used for qualitative and/or quantitative (relative and/or absolute) proteome analysis and identification through mass spectrometry (MS). Lower panel indicates some protein targets (metabolic, contractile, stress-, and signalling related) associated with cardiac hypertrophy or modulated by hypertrophic process. *α*-enolase(P) (phosphorylated alpha-enolase) and cTnL(P) (phosphorylated cardiac troponin I), SCAD (short-chain acyl-CoA dehydrogenase), NADH (nicotinamide adenine dinucleotide), LDH (lactate dehydrogenase), CK (creatine kinase), TPI (triose phosphate isomerase), GSTM2 (glutathione S-transferase Mu 2), ETF-*α* (electron transfer flavoprotein-alpha), Hadha (3-hydroxyacyl-coenzyme A dehydrogenase), NDUFA10 (NADH dehydrogenase (ubiquinone) 1 alpha subcomplex 10), HSP20 (heat shock protein 20), *α*B-crystalline, ANF (atrial natriuretic peptide), MyHC (myosin heavy chain), MyLC (myosin light chain), desmin, and HFABP (heart-type fatty acid binding protein).

In this regard, several experimental models have been used to better understand the effect of hypertension and other pressure overload effects on the cardiovascular system and heart tissue. Moreover, spontaneous development (SHR), transgenic (dTGR: double transgenic rats harbouring human renin and angiotensin genes) and mechanical induction (e.g., aortic constriction) of hypertension are widely used for this purpose [41–43]. Hypertrophic-inducing agents (e.g., ET-1, Ang II, and isoproterenol) are also often used in cardiomyocytes to investigate the molecular mechanism and signalling pathways underlying physiologic and different types [39] of pathological hypertrophy [11]. However, it is observed that several of these studies combine physiological observations with biochemical and genomic data, lacking proteomic information. Proteomic research could therefore provide more insights into the molecular events within the cardiac hypertrophy phenotype. Accordingly, this section will describe some relevant proteomic studies from this perspective. Moreover, proteomic workflow and protein targets associated with cardiac hypertrophy are shown in Figure 2.

As mentioned before, left ventricle hypertrophy (LVH) is a well-known characteristic of cardiac adaptation to pressure overload and an essential criterion of hypertensive heart disease [44]. Studies have shown that the LV proteome in particular is highly altered in this process, even at the early stages of hypertension [45]. The spontaneously hypertensive rat (SHR) is one of the main experimental models of essential hypertension, displaying several characteristics of this pathology, including LVH [46]. In this experimental model, research has highlighted the role of protein phosphorylation as a molecular signature common to the pathogenesis of cardiac hypertrophy [40, 47].

Furthermore, phosphoproteins such as *α*-enolase, SR-Ca^2+^-ATPase, and phospholamban have been shown to be crucially associated with cardiac hypertrophy induced by hypertension in SHR [40, 47]. In this regard, LV proteins from SHR and control (Wistar-Kyoto) rats were enriched for phosphoproteins (phosphoaffinity chomatography column) and then analysed by 2-DE, followed by phosphoprotein specific staining (Pro-Q diamond) identification by MALDI TOF [40]. Here, 21 protein spots were significantly altered between groups where 19 proteins were identified as being related to metabolism, contraction, cell cycle, and signalling. Multiple phosphorylations were also observed, with attention to 3-ketoacyl-CoA thiolase, which had not been previously shown to be phosphorylated. In this study, close attention was paid to the hyperphosphorylation of *α*-enolase in SHR, which was also seen in younger SHR (4 weeks old, data not shown) but was not present in the right ventricle or in atria. Authors have shown that four-week-old SHR did not develop hypertension, indicating that the hyperphosphorylation of *α*-enolase may not be secondary to hypertension. Moreover, in the present study it was shown that *α*-enolase enzymatic activity is reduced by phosphorylation in LV. These data seem to be inconsistent with the literature, where anaerobic glycolysis is shown to be enhanced in several models of cardiac hypertrophy [11, 48]. Thus, it is speculated that hyperphosphorylation of *α*-enolase in LV of SHR may display another function beyond its catalytic activity.

The LVH proteome has also been investigated in two different animal models of hypertension to verify key proteins related to hypertensive hypertrophy [49]. In this study, the LV proteome from SHR (model of essential hypertension), renovascular hypertensive rats (RHR, a model of secondary hypertension made by clipping renal arteries), and control rats (Wistar-Kyoto) was shown to present a distinct proteome profile. Two-D DIGE MALDI TOF detected 29 protein spots with a significant difference in expression (2-fold) among the groups (20 spots between RHR and SHR, 23 between SHR and control rats, and 19 between RHR and control rats). From this total, 18 protein spots were identified belonging to 16 unique proteins (including different isoforms and posttranslational modifications). Moreover, glutathione-S-transferase (GSTM2) and short-chain acyl-CoA dehydrogenase were (SCAD) both downregulated in SHR but not in RHR, compared with control animals; results were confirmed by Western blot, RT-PCR, and enzymatic activity. A different pattern was seen in LVH in both models, which may result from the distinct proteome profile seen in this study, where GSTM2 and SCAD may be relevant candidates in the development of LVH in SHR. Moreover, also it was shown that LVH regressed by pharmacologic means still maintains the proteomic characteristics of hypertrophied hearts [50]. This was shown by 2-DE MALDI-TOF analysis, where 53 protein spots (related to 36 unique proteins) were altered in hypertensive hearts (e.g., upregulation of SCAD, NADH, enolase 1*α*, and aldehyde dehydrogenase and downregulation of ETF-*α*, superoxide dismutase, and thiol-specific antioxidant). The authors showed that antihypertensive treatment led to normalization of proteins related mainly to contractive and stress-related processes, but those 17 proteins with an essential role in energy production, cell stress defence, and hypertrophy regulations remained unchanged after LVH regression.

The role of myocardial K_ATP_ channels in cardiac hypertrophy has been widely investigated to date [51–53]. K_ATP_ channels are ATP-sensitive channels formed by four pore Kik6.2 subunits and four regulatory SUR1 subunits, known to present cardioprotective properties, due to their integration with other myocyte protein channels and proteins associated with cellular bioenergetics pathways, playing a prominent role in metabolic homeostasis [52]. Research has shown that deficiency in myocardial K_ATP_ channels is currently thought to play a role in hypertension pathophysiology [54, 55]. Comparative 2-DE analysis followed nanoelectrospray LC-MS/MS [53], and Orbitrap MS protein identification [51] found that an experimental model lacking Kir6.2 ATP-sensitive K(+) (K(ATP)) channels generates unfavourable cardiac proteome remodelling in hypertensive myocardium. Both studies have shown that over 170 proteins presented a significant differential expression in response to dysfunction of K_ATP_ channels, with 95 proteins being linked with metabolic function (e.g., lactate dehydrogenase, SCAD, pyruvate kinase, triosephosphate isomerase, and creatine kinase), and they are also associated with bioenergetic enzymes that were previously linked to K_ATP_ channel activity in other studies [52]. Thus, because Kit6.2, an isoform of cardiac K_ATP_ channels, is associated with stress adaptation within the myocardium, dysfunction of K_ATP_ channels is thought to underlie heart disease [52, 56].

Proteinases seem also to be a relevant class of proteins in the pathophysiology of hypertension, due to their central role in blood pressure control among other vital physiologic functions such as coagulation [57, 58]. In this way, MS-based proteomics is a robust tool in the research of the complex protease network such as the renin-angiotensin system (RAS), a widely investigated proteolytic network with a central role in hypertension development [59–61]. Moreover, the RAS also acts in a tissue-specific way (e.g., brain, skeletal muscle, kidney, and myocardium), presenting distinct local physiological responses [62]. The heart's local RAS is known to be stimulated by hemodynamic stress (e.g., pressure and volume overload), where angiotensin II is the main vasoactive product of this system, and also known to modulate contractile-related molecular expression (skeletal *α*-actin, *β*-myosin heavy chain, atrial natriuretic polypeptide, and fibronectin) and promote cardiac phenotype remodelling [63] and hypertrophy [41, 64]. Otherwise, inhibition of Ang II by angiotensin converting enzyme inhibitors (ACEI) attenuates cardiac hypertrophy induced by pressure overload in experimental models and humans [62, 65], and it has been established that the inhibition of RAS attenuates and regresses cardiac hypertrophy induced by hypertension [66]. Moreover, Ang II receptors, AT_1_ and AT_2_, have been widely investigated as intermediates for pathological stimuli in the cardiovascular system, where the stimulation of AT_1_ (a *G*-protein-coupling receptor) is shown to trigger vasoconstriction signalling [62, 67] and cardiac hypertrophy through the activation of mitogen-activated protein kinase (MAPK) and protein kinase (PK) [68].

Heart mitochondrial proteome profiling by LC-MS/MS analysis in dTGR (double transgenic rats harbouring human renin and angiotensin genes) after caloric restriction (60% of energy intake for 4 weeks) revealed seven differential proteins compared to dTGT without caloric restriction. Moreover, the present study identified 6 proteins (downregulation of cytoskeletal and enzyme modulators and upregulation of oxidoreductase) present only in dTGR rats compared to the other experimental groups, including Sprague-Dawley rats control group [69]. The present study also indicated that CR attenuated cardiac hypertrophy, fibrosis, and cardiomyocyte apoptosis, suggesting that modulation in the mitochondrial proteome by caloric restriction may attenuate cardiovascular disorders induced by Ang II. Besides proteomic analysis, cardiac hypertrophy induced by Ang II in the dTGR model was also shown by gas-chromatography TOF to modulate the cardiac metabolome in more than 100 metabolites [70]. Moreover, comparative label-free LC-MS/MS analysis revealed that pressure overload heart hypertrophy induced by aortic constriction led to downregulation in the abundance of mitochondrial fatty acid oxidation proteins and upregulation of pyruvate dehydrogenase subunits and tricarboxylic acid cycle proteins [71]. These data sustain the role of RAS components in cardiac remodelling induced by Ang II, as well as the relation between mitochondrial dysfunction, altered cardiac metabolism [70] (e.g., downregulation of mitochondrial and lipid metabolism genes) [72], and the proteome as pivotal factors in cardiac pathological hypertrophy.

Despite the technical difficulty in separating cytosolic from mitochondrial proteins and other contaminants as well as determining the relevant cytosolic proteins which translocate to mitochondria during several physiological processes (e.g., apoptosis) [73], research into the mitochondrial proteome is an important issue in maladaptation in cardiac hypertrophy [3, 45]. Several data indicate mitochondrial dysfunction and impairment in cardiomyocyte metabolism as strong characteristics in overload cardiac hypertrophy [74–76]. Moreover, the altered cardiac mitochondrial proteome was recently shown to precede and contribute to the development of hypertension in spontaneously hypertensive rats [45]. In this study, authors showed by 2D-DIGE combined with MALDI TOF/TOF that prehypertensive (4-week-old rats) and further hypertensive stage (20-week-old rats) harbour distinct mitochondrial proteome in the left ventricle portion. It was observed that the prehypertensive stage presented a greater proteome alteration (significant alteration in 33 protein spots, 16 upregulated and 17 downregulated) compared to the 20-week-old SHR (13 protein spots significantly altered). In this study, the authors highlighted the alteration in mitochondrial trifunctional enzyme alpha subunit (Hadha) and dehydrogenase 1 alpha subcomplex 10 (NDUFA10) as possible relevant molecular agents in the development of cardiac hypertrophy in SHR, since both enzymes were differentially expressed as early as one week of age in this rat strain.

Myocyte hypertrophy is also stimulated by different signalling pathways through the stimulation of endothelin-1 (ET-1), which includes the protein kinase C, phosphatidylinositol 3-kinase, and mitogen-activated protein kinase (MAPK), which also includes p38 mitogen-activated protein kinase and c-Jun N-terminal kinase pathway [77]. Endothelin-1 is a strong vasoconstrictor peptide hormone and stimulator of RAS, which is widely used to induce cardiac hypertrophy [78]. Recently, 2-DE followed by LC ESI-MS/MS analysis revealed that concentric cardiac hypertrophy induced by ET-1 revealed a distinct proteome compared to eccentric induced hypertrophy [43]. Authors found that twelve different proteins were differently expressed in cardiomyocytes treated with ET-1 compared to control nontreated cardiac cells, where eight proteins were upregulated and another three downregulated. From those, *α*B-crystalline, associated with cardioprotection and ANP, a biomarker for pathologic cardiac hypertrophy, presented the highest upregulations [43]. A more recent study found similar data, indicating that cardiomyocyte hypertrophy induced by ET-1 led to proteome modulation with the increase in expression of desmin protein species and *α*B-crystalline  [79]. Other cardiac hypertrophic stimuli, such as isoproterenol (ISO), were also observed to promote an alteration in healthy cardiac tissue and in the cardiac proteome, shown by 2-DE MS/MS analysis [80]. Isoproterenol is a catecholamine widely applied in cardiovascular research as a model for adrenergic stimulation with a close association with pathological cardiac hypertrophy [81]. Here, seven proteins were differentially expressed in pathological hearts where myosin light chain 2 and 3, desmin, prohibitin, heart fatty acid binding protein, and ATP-synthase 5*β* were downregulated, while heat shock proteins 60, 70, and D1 were upregulated. Although some data have been shown to be contrary to previously reported studies (e.g., desmin upregulation shown by Agnetti et al. [79]), this may indicate a variation in cardiac response to something other than cardiac hypertrophy stimulus (e.g., ISO versus ET-1 hypertrophic stimuli).

Finally, the transition from pathological hypertrophy to HF makes the discovery of biomarkers for early disease treatment of HF an urgent necessity. Troponin I seems to present a high specificity for this purpose. Analysis of pathological and healthy human heart tissue by top-down MS-based quantitative proteomics has detected the phosphorylation of cTnI in Serine 22/23 sites at an early stage of CHF, making it a strong candidate biomarker for this pathologic state [82]. This study also presents top-down proteomics as a viable clinical tool in biomarker research. Moreover, the investigation of the molecular mechanisms involved in pathological hypertrophy is of great interest due to the high correlation with heart failure [26]. Although the entire molecular mechanisms underlying the development of pathological heart hypertrophy have not been fully elucidated, it has been noted that this process is coordinated by multifactorial events rather than by a single target or stimulus. Furthermore, pharmacologic and alternative strategies such as exercise may be addressed to prevent and treat pathological cardiac hypertrophy. The main alterations in cardiac proteome listed in this section are presented in Table 1.

## 5. Proteomic Research in Other Target Tissues 

Rapid advances in the genomic field have led to large amount of data in hypertension research, ranging from the analysis of several candidate genes to high-throughput genetic mapping (e.g., complex genome-wide scans) [5, 6]. Moreover, it has been seen that the genomic approach is likely to investigate the pathogenesis of hypertension rather than its pathophysiology [36]. Functional genomic analysis, and more recently, proteomics, have both been widely used to better understand the pathophysiology of hypertension. In this regard, the main advance of proteomics, as a postgenomic tool, is its ability to identify gene products, PTM, and further investigate the expression of these protein species for phenotype and physiological responses [37]. Undoubtedly, proteomic analysis plays an important role in hypertension research, where the cardiac and vascular proteomes have been the main focus [25, 36, 82–86].

Several proteomic studies involving the pathophysiology of hypertension have been carried out in renal and vascular tissue. Among these, Thongboonkerd et al. [87] performed an elegant study evaluating the effect of hypoxia (a component of obstructive sleep apnoea, closely associated with hypertension) on the renal proteome in Sprague-Dawley rats. In this study, rats submitted to intermittent hypoxia developed hypertension, while 2-DE analysis indicated changes in protein involved in the renal kallikrein system (kallistatin and A1AT) and regulation of vascular hypertrophy. In contrast, rats submitted to sustained hypoxia presented an upregulation of b2-bradykinin receptor and elevated kallikrein levels with normalized levels of blood pressure not developing hypertension. These data suggest that these alterations in the renal proteome in response to sustained hypoxia are related to a compensatory effect in vasodilation and vascular remodelling in order to prevent the development of hypertension. In another study using classic 2-DE analysis, Pinet et al. [88], using a two-kidney, one-clip method in Goldblatt rat model or renovascular hypertension, found that troponin T decreased in renal arterioles from the clipped kidney, indicating this protein as a possible biomarker in the pathophysiology of renovascular hypertrophy.

The urinary proteome has also attracted much interest in hypertension research, because clinical proteomics aims to detect possible biomarkers for left ventricle diastolic dysfunction and diastolic heart failure (associated with hypertension). Moreover, the urinary proteome may represent an advance in the early diagnosis of hypertension. In this context, Kuznetsova et al. [83] used capillary electrophoresis coupled with mass spectrometry (CE-MS) to screen and identify peptides and polypeptides (collagen polypeptides) that might be associated with the early stage of left ventricle dysfunction in hypertensive patients. After initially identifying 85 potential biomarkers (*P* < 0.033), the authors identified three polypeptides (collagen alpha-1(V), WW domain-binding protein 1,1 and isoform 1 of collagen alpha-1) that were significantly downregulated in patients with LV dysfunction compared to control patients. However, the authors also stated the need for larger cohort studies to better establish the accuracy of using the urinary proteome to identify new biomarkers in LV dysfunction. The urinary proteome has also been used to identify conditions associated with hypertension, such as preeclampsia renal injury [89–91] and other pathologies associated with hypertension such as diabetes [92].

In addition, the relationship between hypertension and arterial thrombosis was investigated by analysing the platelet proteome by 2-DE [93] in two distinct rodent models of induced hypertension (cyp1a1ren-2 transgenic rats fed with indole-3-carbinol and Fischer 344 rats induced with subcutaneous infusion of angiotensin II). In this study, 45 proteins spots were shown to be altered during hypertension induction in both animal models, and the expression of all protein spots was reversed after 10 days of blood pressure normalization. Moreover, the authors identified by mass spectrometry 38 spots that were assigned to 20 proteins (mainly protein fragments), which indicate that hypertension induced by angiotensin II may be associated with protein degradation in platelets. The reversible aspect in this proteome study has led to the prospect of identifying and developing possible novel biomarkers.

## 6. Does Exercise Extenuate Cardiac Pathological Hypertrophy?

Cardiomyocyte plasticity plays an important role in heart adaptation and maladaptation to external stimuli such as pregnancy, exercise, chronic pathology, and genetic disorders. As mentioned during this review, cardiac remodelling is a complex phenotype modification resulting from adverse external and intrinsic stimulus followed by alternative inner cell signalling, gene regulation, and cardiac proteome modulation [11, 94, 95]. In this context, physiologic and pathologic hypertrophy display a distinct molecular mechanism, also confirmed by proteomic data [11, 94]. In the previous section, several proteins related to metabolism, myocyte contraction, and stress response were shown to be altered in pathological hypertrophy, especially in LV. Thus, these proteome modulations were associated with the altered metabolism, fibrosis, and contractive dysfunction seen in hypertensive hearts [49, 50]. Lastly, pathological hypertrophy is characterized as an irreversible process.

Contrarily, physiologic cardiac hypertrophy in response to pregnancy and chronic exercise is a reversible process and associated with improvement in cardiac function and increased heart resistance to ischemic insult [11]. Exercise stimuli have been extensively shown to modulate the heart proteome [94, 96–102] which is normally followed by an improvement in aerobic capacity [98]. Furthermore, improved aerobic capacity is an independent factor for health status, being also inversely correlated with cardiovascular diseases [103], with exercise being a strong factor for preventing and treating hypertension and associated pathologies such as obesity and diabetes [104]. Moreover, exercise is a nonpharmacologic agent and the main choice for hypertension treatment among other cardiovascular diseases, such as heart failure and myocardial infarction [1].

The role of exercise stimulus in blood pressure (BP), endothelial function, and cardiac hypertrophy in the experimental model is still under debate [105], where exercise intensity seems to be a key factor in this process [106]. Research has shown that low exercise training attenuates systolic hypertension and improves mitochondrial status and contractile dysfunction, delaying heart failure in a hypertensive experimental model [16, 107]. While moderate exercise (70% of maximal running speed) did not affect BP, it did not worsen cardiac function in severe hypertensive rats induced by renal artery constriction (two-kidney, one-clip model) [18]. Moreover, exercised hypertensive rats (SHR) were shown to present reduced levels of BP compared to sedentary SHR, while exercised SHR and sedentary nonhypertensive rats (Wistar) presented a reduced aorta wall thickness compared to sedentary SHR [108]. Exercise has also been shown to correct abnormal Ca^2+^ handling in heart failure rats [14], attenuate systolic dysfunction, and improve bad phosphorilation (e.g., pro-apoptotic molecule) in the early stage of hypertension, independent of relieving apoptosis [107]. Exercise training was also shown to superimpose hypertension impacts on LV remodelling, increasing cardiomyocyte length and width to a greater degree than in nontrained SHR, also attenuating apoptosis [109]. Cardiac mitochondrial apoptotic signalling was also shown to be reduced by aerobic exercise in an obese animal model with prehypertensive BP status [110]. On the other hand, it has been shown that high exercise intensity may be considered a risk factor in the hypertensive phenotype rather than a therapeutic factor, since this intensity was shown to accelerate hypertensive effects and improved fibrosis in SHR [111]. Despite these data from the literature, there is still a need for more proteomic data concerning the effect of exercise on the pathological heart to better understand the effects of exercise on pathological hypertrophy induced by hypertension.

Concerning the metabolism status, it has been demonstrated that rodents with natural inborn low aerobic capacity harbour an altered and perturbed energy metabolism and an enhanced oxidative stress in heart proteome [112]. Contrary to the metabolic dysfunction (e.g., reduction in FFA oxidation) seen in hypertensive hearts, endurance exercise is associated with improvements in cardiac metabolic enzymes, especially in fatty acid oxidation as reviewed by Burniston and Hoffman [94].

In this regard, the expression of several metabolic enzymes (short-chain acyl-CoA dehydrogenase and enzymes from the *β*-oxidation TCA cycle) from LV was shown to be altered after high intensity swimming [102]. Swimming training also led to cardiac hypertrophy in nonpathological rats. Further research in LV proteome showed that moderate treadmill running led to diverse alteration in the contractile, stress-related, and metabolic function of cardiac proteins, where heart fatty acid binding proteins (HFABP), thioesterase-1, and short-chain acyl-CoA dehydrogenase were upregulated [98]. Moreover, one single bout of high intensity swimming at moderate and high intensity was also shown to modulate LV proteins from obese (ob/ob) and control nonobese mice (ob/OB) [100]. However, in this study, HFABP was downregulated after high intensity exercise in nonobese mice but not in obese mice. Moreover, aspartate aminotransferase, an analogue of plasma membrane fatty acid transporter (FABPpm), was also upregulated in nonobese mice, possibly indicating an acute uptake of long-chain fatty acids. In this study, mitochondrial aconitase was downregulated in both rodent phenotypes, while HFABP was downregulated only in obese mice. In a recent study, the LV proteome from Wistar rats was shown to be modulated, following different swimming exercise intensities adjusted according to each animal's body weight [101]. Moderate and high intensity resulted in the upregulation of contractive proteins, mainly *α*-MHC (alpha-myosin heavy chain) and troponin accompanied by cellular injury in the high intensity group. The metabolic enzyme, NADH dehydrogenase, was also differentially expressed in response to high exercise intensity. Although high intensity was associated with greater proteome changes, this intensity was associated with cardiac cell damage compared to low and moderate intensities. Alteration in contractile, metabolic, and mitochondrial enzymes induced by endurance exercise occurred in an opposite way from the changes seen following pressure overload pathological hypertrophy and heart failure [71].

In an ischemia/reperfusion experiment, exercise training was shown to alter cardiac mitochondrial proteins and protect the heart against IR-induced myocardial damage, also by presenting an antiapoptotic effect [99]. In research using isobaric tags for relative and absolute proteome quantitation (iTRAQ), authors identified 222 mitochondrial proteins, where 13 were significantly altered by endurance training (8 upregulated and 5 downregulated). Moreover, downregulation of mitochondrial proteins, MAO-A (monoamine oxidade) and PRDXIII were identified as novel potential candidates of exercise-induced cardioprotection since they play a prominent role in oxidative stress and apoptosis, with MAO-A being associated with pressure overload pathology hypertrophy and heart failure [113, 114]. Moreover, relative and absolute proteome quantitation have significantly improved proteomic investigation in several areas including cardiovascular research [115–117]. More recently, endurance exercise was shown to play a positive role in cardiac function after myocardial infarction [96]. Two-DE analysis revealed that exercise training induced the upregulation of glutathione peroxidase-1 and manganese superoxidase dismutase, with both being related with antioxidative activity induced by exercise [118].

Lastly, heat shock protein 20 is a widely researched chaperone due to its role in cardioprotection [119, 120]. Boluyt et al. [97], in the first study involving exercise and the cardiac proteome, demonstrated that six weeks of endurance training led to adaptive cardiac hypertrophy and significantly altered 26 protein spots in LV, where 12 spots, including the HSP20, were exclusive to trained rats. Authors also showed that the expression of shp20 only followed exercise training rather than a single bout of exercise. Furthermore, shp20 was also shown to be upregulated in Wistar rats following moderate exercise endurance (75% of VO_2max⁡_), compared to sedentary animals [98]. The proposed exercise program was shown to increase cardiac mass (11%) and to improve animals' aerobic capacity (VO_2max⁡_ increase by 23%). In this study, MS/MSA revealed that exercise-induced shp20 is phosphorylated at Serine 16 in a few hours after exercise. Again, protein phosphorylation may be associated with a cardioprotection process, since the blockade of HSP20phosphorylation is shown to enhance ischemia/reperfusion injury [121].

Despite the scarcity of proteomic research performed with exercise and heart tissue, the present data indicated that the altered proteome is mostly associated with cardioprotective aspects such as contractile and metabolic improvement and physiologic cardiac hypertrophy. Moreover, the degree of cardiovascular adaptation to exercise is intensity dependent, where, as previously shown, high intensity exercise may enhance hypertensive stimulus [111] and be associated with cardiac damage [101]. Thus, it is suggested that more research should be performed, taking into account the effect of different types and intensities of exercise on the heart proteome.

## 7. Conclusions and Prospects 

The various advances in high-throughput platforms have led to multianalysis of genes, proteins, and other molecular components that may be involved in hypertension pathogenesis and pathophysiology. Therefore, despite progress in proteomic research, the multifactor aspect of hypertension still needs to be explored by a multiplex strategy, which certainly involves a number of other “omics” tools and analysis strategies such as those seen in systems biology. In this view novel techniques in addition to classical proteomics tools including mass spectrometry- (MS-) based proteomics, posttranslational modifications detections, and next-generation sequencing (NGS), which are fast maturing procedures, are enabling comprehensive measurements of gene products at a system of hypertension pathogenesis and pathophysiology level [122]. Although MS and NGS are extremely complementary, they are still rarely applied and integrated in large-scale studies including exercise and hearth pathology. Nevertheless, all those techniques must also apply together in order to shed some light on those important and complex systems. Technological advances in both the proteomics and transcriptomics community also may offer the capability to distinguish genetic and posttranscriptional polymorphisms at the proteome level. These advances also allow improved gene expression quantitation, which is restricted by the imprecise proxy of transcriptome data alone. In summary authors believe that synergistic utilization of multiple techniques including genomic, transcriptomic, and proteomic technologies will significantly improve information, enhancing proteogenomics to a top level in exercise and hypertension studies. The main challenges in cardiac proteomic and hypertensive research and the future directions on this field are presented in Table 2. Such actions are remarkable challenges for the next years and could, in our opinion, clearly contribute to development of cardiac and hypertension proteomics.

## Figures and Tables

**Table 1:** Cardiac proteome modulated by pathologic cardiac hypertrophy.

Experimental model	Experimental method	Main altered proteome	Reference
SHR and WKY	2D-DIGE	Comparison between different SHR age and animal models: (i) 33 mitochondrial proteins with altered expression between SHR groups; (ii) Hadha and NDUFA10 with differential patterns in SHR versus WKY.	[45]

SHR	Phosphoaffinity chromatography; 2-DE; Pro-Q staining; MALDI TOF	Protein phosphorylation in cardiac hypertrophy linked with hypertension: (i) 3-ketoacyl-CoA thiolase; (ii) *α*-enolase hyperphosphorilation (reduced enzymatic activity); (iii) SR-Ca^2+^-ATPase and phospholamban.	[40, 47]

Human heart tissue	Top-down MS-based quantitative proteomics	Phosphorylation of cTnl in Serine 22/23 as candidate biomarker of CHF.	[82]

SHR versus RHR and WKY	2D-DIGE/MALDI TOF	Comparison between two distinct models of heart hypertrophy: (i) 29 protein spots with differential expression among the three groups (18 proteins identified); (ii) ↓ GSTM2 and SCAD in RHR versus WKY; (iii) Distinct profile of GSTM2 and SCAD between SHR and RHR.	[49]

WKY	2-DE/MALDI TOF; Pharmacologic treatment	Effect of pharmacologic treatment over LVH regression: (i) 36 proteins altered in hypertensive heart; (ii) ↑ SCAD, NADH, enolase 1*α*, and aldehyde dehydrogenase; (iii) ↓ ETF-*α*, superoxide dismutase, and thiol-specific antioxidant.	[50]

Animal model lacking Kir6.2 ATP-sensitive K(+) (K(ATP)) channels	2-DE; LC-MS/MS; Orbitrap MS	Deficiency in myocardial K_ATP_ channels and hypertension pathophysiology: (i) 170 proteins with differential expression in response to K_ATP_ channel dysfunction; (ii) LDH, SCAD, pyruvate kinase, TPI, and CK.	[51–53]

Animal model and human	Transcriptome	Proteinases and the pathophysiology of hypertension: (i) Induction of cardiac hypertrophy by Ang II; (ii) Attenuation of cardiac hypertrophy by Ang II and RAS inhibition.	[41, 62–66]

dTGR and Sprague-Dawley rats	LC-MS/MS	Caloric restriction in dTGR over mitochondrial proteins: (i) 7 differential proteins after caloric restriction in Dtgr; (ii) 6 proteins unique to dTGR compared to caloric restricted dTGR and SD rats; (iii) ↓ 6 proteins (cytoskeletal and enzyme modulators) and ↑ oxidoreductase.	[69]

dTGR	Gas-chromatography TOF	Cardiac hypertrophy induced by Ang II: Modulation of >100 cardiac metabolites.	[70]

Aortic constriction in rodent model	Label-free LC-MS/MS	Pressure overload cardiac hypertrophy: (i) ↓ mitochondrial fatty acid oxidation proteins; (ii) ↑ pyruvate dehydrogenase subunits and TCA proteins.	[71]

Animal model and cell culture	2-DE; LC ESI-MS/MS	Cardiac hypertrophy induced by ET-1 and leukemic inhibitory factor exposure: (i) Differential proteome between ET-1 (concentric) and eccentric induced hypertrophy; (ii) ↑*α*B-crystalline in nontreated cells; (iii) ↑ ANP upregulated in both cardiac hypertrophy models; (iv) ↑ desmin protein species.	[43, 79]

Animal model and cell culture	2-DE MS/MS	Cardiac hypertrophy induced by ISO: (i) 7 differential expressions in heart induced with ISO; (ii) ↓ MLC 2 and 3, desmin, prohibitin, FABP-H, and ATP-synthase 5*β*; (iii) ↑ HSP60, 70, and D1.	[80, 81]

ANP: atrial natriuretic polypeptide; CHF: chronic heart failure; CK: creatine kinase; cTnl: cardiac troponin; dTGR: double transgenic rats harbouring human renin and angiotensin genes; ET-1: endothelin-1; ETF-*α*: electron transfer flavoproteins-*α*; FABP-H: heart fatty acid binding protein; GSTM2: glutathione-S-transferase; Hadha: mitochondrial trifunctional enzyme alpha subunit; HSP: heat shock proteins 60, 70, and D1; ISO: isoproterenol; LDH: lactate dehydrogenase; LVH: left ventricle hypertrophy; MLC 2 and 3: myosin light chain 2 and 3; NDUFA10: NADH dehydrogenase 1 alpha subcomplex 10; RHR: animal model of secondary hypertension performed by clipping renal arteries; SCAD: Short-chain acyl-CoA dehydrogenase; SHR: spontaneously hypertensive rat; TCA: tricarboxylic cycle; TPI: triosephosphate isomerase; WKY: Wistar-Kyoto; *β*-MHC: *β*-myosin heavy chain.

**Table 2:** Challenges and future perspectives in cardiac proteome in hypertension research.

Challenges in cardiac proteomic and hypertension research:	
(i) integration of “omics” tools as a multiple strategy;	
(ii) MS-based proteomics coupled with NGS approach;	
(iii) proteomic and genomic large-scale studies in hypertension development and treatment;	
(iv) identification of posttranslational polymorphism and genetic factors;	
(v) identification of novel differential molecular signalling and expression between physiologic and pathologic cardiac hypertrophy;	
(vi) identification of novel hypertension biomarkers in blood samples.	

Future direction in cardiac proteome and hypertension research:	
(i) novel studies cross talking proteomic and genomic data;	
(ii) improvement in gene expression quantitation and transcriptome data;	
(iii) identification of novel pharmacologic targets and nonpharmacologic strategies in hypertension attenuation;	
(iv) novel drug design and texting in cellular and experimental hypertensive models;	
(v) investigation of exercise and other alternative strategies in hypertension attenuation.	

NGS: next generation sequencing.
